# Isopathy of the Past and Future

**Published:** 1884-01

**Authors:** 


### ISOPATHY OF THE PAST AND THE FUTURE.

Culpeper's *London Dispensatory* was in its day a famous work; from it a few paragraphs are quoted to give one some idea of the isopathy of the past. By the way, Dr. Culpeper signs himself "Nich. Culpeper, Gent. Student in Physick and Astrology," showing how medicine and astrology were practiced together in former days. But we can scarcely relegate this practice exclusively to *former* times, for have we not in these latter days preparations of "Sol," "Luna," etc.!!

At page 30, of the old edition, we find some remarks on the use of "living creatures" in medicine. Thus: "The flesh of vipers being eaten, cleer the sight, help the vices of the nerves, resist poison exceedingly; neither is there any better remedy under the Sun for their bitings than the head of the Viper that bit you, bruised [triturated?] and applied to the place, and the flesh eaten, you need not eat above a drachm at a time,* and make it up as a troches of Vipers. Neither any comparable to the stinging of Bees and Wasps, &c., than the same that stung you, bruised and applied to the place."†

"Land Scorpions cure their own stingings by the same means, the ashes of them (being burnt) potently provokes the urine and breaks the stone."

So much for the isopathy of the past. It was a crude thing and possibly of little service, though its adherents doubtless boasted most confidently of its value. To the isopathy of the future we turn with the greatest expectancy; before it lie the grandest achievements, for through its beneficent powers immortality itself will be reached. And in this way. In Salmon's *New London Dispensatory* (A. D. 1678) we are told how to prepare the "Aqua Divina." Thus, "Take the whole carkess of a man violently killed, with the Intrails, cut in pieces, and mix [*i. e.*, triturate] them, distil it from a retort,‡ twice or thrice." "It is reported to have a Magnetic power."

Now, we all know that "potentization makes the medicine homœopathic," *therefore*, all we need to do is to properly potentize this *aqua divina*, and, lo! we possess a remedy homœopathic to death itself! (Who will *dare* say the "Wandering Jew" is *not*

---

*We fear Dr. Culpeper was a member of the American Institute, and believed in using doses that one could *see*, *taste*, and even *smell*. The practice is reprehensible.

† Topical applications are injurious, *vide* I. H. A. resolution No 11,822.

‡ By "retort," our forefathers probably referred to a F. C. potentizer.

an isopathist, and *hence* the longevity of his days?) What an imposing and beneficent role will this isopathist of the future play! See him as he solemnly and yet confidently enters the chamber of death; on the bier lays the dead, around it gather the sorrowing relatives and sad friends. Slowly affixing his potentizer to the nearest cold water spicket, the isopathist promptly potentizes a piece of the dead man's flesh. When this crude mortal substance—a product of disease—is duly raised to the immortal, ethereal sphere of DCM, a drop is carefully placed on the cold tongue of the deceased. The effect is magical. The stilled heart once more pulsates, the inactive nerve centres again send forth their electric commands, the palsied muscles once more are active, the deceased arises, and sorrow is turned into joy. The doctor gets his fee; the undertaker, his dismissal; the heirs, nothing!

Then, indeed, may the isopathist exclaim, in the beautiful words of St. Paul:

> "O death! where is thy sting?
> O grave! where is thy victory?"

